# International Study of Movement Behaviors in the Early Years (SUNRISE): Results from SUNRISE Sweden’s Pilot and COVID-19 Study

**DOI:** 10.3390/ijerph17228491

**Published:** 2020-11-16

**Authors:** Christine Delisle Nyström, Christina Alexandrou, Maria Henström, Ellinor Nilsson, Anthony D. Okely, Serina Wehbe El Masri, Marie Löf

**Affiliations:** 1 Department of Biosciences and Nutrition, Karolinska Institutet, NEO, Group MLÖ, 141 83 Huddinge, Sweden; christina.alexandrou@ki.se (C.A.); maria.henstrom@ki.se (M.H.); serinawehbe@hotmail.com (S.W.E.M.); marie.lof@ki.se (M.L.); 2 Department of Health, Medicine and Caring Sciences, Linköping University, 581 83 Linköping, Sweden; ellinor.nilsson@liu.se; 3 Early Start, Faculty of Social Sciences, University of Wollongong, Northfields Ave, Wollongong, NSW 2522, Australia; tokely@uow.edu.au; 4 Illawarra Health & Medical Research Institute, Wollongong, NSW 2522, Australia

**Keywords:** COVID-19, moderate-to-vigorous physical activity, movement behaviors, preschool children, screen time, sleep

## Abstract

The International Study of Movement Behaviors in the Early Years (SUNRISE) was initiated in response to the 2019 WHO guidelines for physical activity, sedentary behavior, and sleep in children aged 0–5 years. This Swedish pilot study aimed to: (i) assess the proportion of preschoolers meeting the guidelines, (ii) evaluate the feasibility of the methods for the SUNRISE study, and (iii) assess how movement behaviors have been affected in preschoolers during the COVID-19 pandemic. Physical activity and sleep (waist-worn ActiGraph); screen time and movement behaviors (parental questionnaire); motor skills (Ages and Stages Questionnaire); and executive functions (3 iPad games) were assessed in 100 Swedish preschoolers (*n* = 58 boys). There were 19.4% of preschoolers (*n* = 14) who met the WHO guidelines. The motor skill and executive function assessments were feasible; however, 20% refused to wear the ActiGraph overnight. Additionally, during the pandemic Swedish children’s physical activity, time spent outside on weekdays and weekend days, and screen time significantly increased (+53; +124; +68; +30min/day, respectively, all *p*-values ≤ 0.001). Methods for the SUNRISE study were feasible in a Swedish context; however, considerations to switch to a wrist-worn accelerometer should be made. Furthermore, children’s physical activity increased during the pandemic, which is likely due to how the rules/restrictions were implemented in Sweden.

## 1. Introduction

Childhood overweight and obesity represents one of the most significant public health challenges of the 21st century, with around 38 million children under five years of age being classified as overweight or obese in 2019 [1]. More specifically, in Sweden, in 2018 approximately 12% of four-year-old children were classified as overweight or obese [2,3], with this figure almost doubling when children reach 10–11 years of age [4,5]. This is concerning as children with obesity often have co-morbidities such as hypertension, early cardiovascular disease markers, insulin resistance, and psychological consequences and are at a greater risk of premature death, disability and obesity in adulthood [1].

Movement behaviors encompass physical activity, sedentary behavior (inclusive of screen time) and sleep and it is well established that physical inactivity, increased sedentary time, and insufficient sleep are factors contributing to the development of overweight and obesity in childhood [6]. To date, these behaviors and their health implications in children aged zero to five years have primarily been investigated separately [7]. This is flawed since the amount of time spent in one movement behavior will depend on the composition of the rest of the behaviors due to the 24 h continuum. For instance, increasing screen time may decrease physical activity and/or sleep. Thus, there is a need for an integrated approach to assess all three movement behaviors, which was first acknowledged by Canada in 2017 when Tremblay et al. [8] created the Canadian 24-Hour Movement Guidelines for the early years with Australia developing similar guidelines soon after [9]. In April 2019, the World Health Organization (WHO) released integrated global guidelines on physical activity, sedentary behavior, and sleep for children under five years of age [10].

With the newly developed integrated movement guidelines from the WHO [10] comes a need to assess the proportion of children worldwide who are adhering to these guidelines. The International Study of Movement Behaviors in the Early Years (SUNRISE) was initiated and includes countries of varying economic status and pilot work for this study began in 2018. The aims of the main SUNRISE study are to assess the proportion of children meeting the WHO Global guidelines (primary), to determine how movement guidelines are associated with important health, learning, and developmental outcomes in the early years, and to examine variations among low-, middle- and high-income countries [11]. Therefore, before the main study can be initiated, pilot work must be conducted to assess that the methods are feasible in each country and this manuscript reports the results from the Swedish pilot study.

The coronavirus disease (COVID-19) first emerged in late 2019 and was declared as a pandemic in March 2020. Countries have faced challenges with how to handle the crisis, with national governments all responding in different ways. In Sweden, high schools as well as universities/colleges were closed and all education was performed via distance learning. In contrast to many countries, however, grade school as well as preschool/childcare centers remained open. Strict rules were put in place where children were not allowed to attend preschool if they were symptomatic and had to be at home for at least two days without symptoms before they were allowed to return to preschool. Even though preschools remained open, not all parents sent their children to preschool and therefore, movement behaviors may have been greatly affected in these children. Data regarding movement behaviors was recently collected in the Swedish SUNRISE pilot study in the spring of 2019, which provided a unique opportunity to re-assess if and how their behaviors changed in response to the COVID-19 pandemic in Sweden one year later in the spring of 2020.

Thus, the aims of this study were to: (i) assess the proportion of Swedish preschool-aged children meeting the WHO Global guidelines for the early years, (ii) evaluate the feasibility of the methods for the SUNRISE study in the Swedish context, and (iii) assess how movement behaviors have been affected in Swedish preschool children during the COVID-19 pandemic.

## 2. Materials and Methods

### 2.1. Study Design and Participants

Twelve of the thirteen preschools that were asked to participate in this study agreed to take part. Eight preschools were from Stockholm County (*n* = 51) and four were from the County of Östergötland (*n* = 49). Of all preschools included in this study there were 176 eligible children with a total of 101 agreeing to partake in this study. One participant withdrew from the study (reason not provided) and therefore the final sample included 100 children (58 boys and 42 girls).

Data collection for the SUNRISE Sweden pilot study occurred between March and May of 2019. Briefly, the director of each preschool was contacted and informed about the study. When a preschool consented to participate, parents of eligible children were contacted. To be included in this study children had to be between three and five years of age and their parents had to be able to read Swedish sufficiently well to understand the study and provide written informed consent as well as complete the parental questionnaire. The parental questionnaire included basic demographic information on the child and parent as well as questions regarding the child’s movement behaviors and was filled out by one parent prior to the measurements in the preschool. When consent was obtained from both the preschools and parents, trained research staff visited the preschools to measure height and weight, gross and fine motor skills, as well as executive functions. A quiet room away from the main room at the preschool was used for the measurements. The room was divided into two sections, with two children being measured at the same time by two trained data collectors. The three executive function tasks were always separated by another task (e.g., gross motor skills, fine motor skills, or anthropometrics) as per the SUNRISE protocol version 5.3. At the end of each measurement the child was fitted with an ActiGraph accelerometer to be worn for three consecutive days.

In May/June 2020 the participating parents in the SUNRISE Sweden pilot study were re-contacted by text message asking if they would be willing to take part in a questionnaire, conducted over the phone assessing how their child’s movement behaviors have been affected by the COVID-19 pandemic. A total of 82 (from the original 101) parents agreed to participate in this questionnaire.

Parents provided informed consent for their child to participate in the study and the study was conducted in line with the Declaration of Helsinki. The SUNRISE Sweden pilot study was approved by the Swedish Ethical Review Authority (2018/346-31; 2020-02392, COVID-19 questionnaire).

### 2.2. Measures

#### 2.2.1. Physical Activity, Sedentary Behavior, and Sleep

The ActiGraph wGT3x-BT tri-axial accelerometer (ActiGraph, Pensacola, FL, USA) was worn over the right hip and was used to assess the children’s physical activity and sleep for two consecutive 24 h periods. The monitor was fitted directly after the measurement at the preschool and was worn for three consecutive weekdays in order to obtain two complete 24 h periods of data. The device was only supposed to be removed for water-based activities (i.e., swimming or showering/bathing). To be included in the analyses the child needed to have at least one valid day of data. The ActiLife software (version 6.1.2.1, ActiGraph Corporation, Pensacola, FL, USA) was used to initialize, download, and process the ActiGraph data. Accelerometers were programmed to capture data at a sampling rate of 30 Hz, which was integrated into 15 s epochs for the analysis using the low frequency filter. The identification of valid 24 h days was done through visual inspection of the ActiGraph data to confirm if there were movement peaks throughout the entire day (defined as midnight to midnight). If there were little to no peaks throughout the sleeping period, it was assumed that the child took off the monitor and was not included in the analyses. Greater than 20 min of consecutive zeros was the criteria used to identify and exclude non-wear time during the time period from 05:00 to 23:30. The cut-points by Pate et al. [12,13] were used to classify different intensities of physical activity.

Nocturnal sleep periods were determined using visual inspection of the 60-s epoch ActiGraph data. To identify bedtime and wake-time a change in the inclinometer output from sitting/standing position to a lying down or off position or vice versa was identified around the parent-reported bedtime or wake time as reported in the parental questionnaire. Bedtime was identified as the first minute followed by 10 consecutive minutes with a vector magnitude of 0 and at least 90 min needed to elapse between bedtime and wake-time [14,15]. Wake-time was identified by at least 10 consecutive minutes with a vector magnitude greater than zero [14,15]. A total of eight children refused to wear the ActiGraph and 20 children refused to wear the ActiGraph overnight or forgot to put the ActiGraph on and were excluded from the analyses examining the proportion of children meeting the WHO Global guidelines.

#### 2.2.2. Screen Time

Screen time was assessed in the parental questionnaire using the following questions: “On a 24-h period in the past week, on days that you spent with your child (i.e., they did not go to childcare/preschool and you were not at work) how much time did your child, who is participating in this study, spend using an electronic screen device such as a smart phone, tablet, video game, or watch television or movies, or videos on the internet while they were sitting or lying down?”.

#### 2.2.3. Anthropometrics

The weight and height of the children were collected using a digital scale (SECA 878) and portable, wall stadiometer (SECA 213) when the children were wearing light clothing and no shoes. The values were rounded to the nearest 0.1 kg or cm, respectively. Body mass index (BMI) was calculated as weight in kilograms divided by height in meters squared. The WHO’s reference standards [16] were used to classify the children into BMI prevalence thresholds.

#### 2.2.4. Executive Functions

Executive functions (i.e., cognitive flexibility, inhibition, and visual-spatial working memory) were assessed using three validated games for the iPad (www.eytoolbox.com.au) [17]. The “Card Sort” task assesses cognitive flexibility. The child is presented with cards that vary along two dimensions (i.e., shape and color) and are asked to sort each card by one dimension (e.g., color) and then by the other dimension (e.g., shape). The total score can range between 0–12 points [17]. The “Go/No-Go” task is an assessment of inhibitory control, i.e., the ability to control behavioral urges and impulses. The child is presented with a fish and a shark and is instructed to tap the iPad screen when a fish appears and refrain from tapping the screen when a shark appears. The score for this task represents the proportion of go trials (catching the fish) multiplied by the proportion of no-go trials (refraining from catching the shark). The score produced is a percentage of accuracy score that ranges between 0 and 1, with 1 referring to a “perfect” score [17]. The “Mr. Ant” game examines the child’s visual-spatial working memory, i.e., the amount of visual information that can be actively maintained. This task was scored by a point which was awarded for every completed level (placing of the stickers correctly two out of three times). Furthermore, an additional third of a point was awarded for all of the correct trials thereafter and the score could range between 0–8 points [17].

#### 2.2.5. Gross and Fine Motor Skills

The Ages and Stages Questionnaire (48 months) [18] was used to assess the children’s gross and fine motor skills. For the gross motor tasks, the child received two attempts and were classified into three categories: “yes”, “sometimes”, or “not yet”. If the child completed the task on the first attempt they were scored as “yes” and if the task was not completed they received another attempt directly after the first attempt. If the task was completed on the second attempt the child was classified as “sometimes” and if they could not complete the task they received “not yet”. The task of climbing up the rungs of a ladder of a slide was not performed as it was not feasible to conduct in all of the participating preschools.

The fine motor skills were performed only once and the children were classified as either “yes” or “not yet”. The exception was for the copying of shapes and drawing of the person where the child was scored as “yes” if they copied or drew ≥3 shapes or body parts, respectively; “sometimes” if they copied 1–2 shapes and include two body parts, respectively; and “not yet” if the child could not complete either task.

Scoring for the Ages and Stages Questionnaire comprises receiving 10 points for “yes”, 5 points for “sometimes”, and 0 points for “not yet” for each task and from that, a total score for both gross and fine motor skills was computed. As one task for gross motor skills was unable to be completed, an adjusted gross motor skill score was calculated. An adjusted score was also calculated if a child refused to participate in a task. Based on their score the children were either classified as below the cut-off (i.e., the child is either at risk or developmentally delayed) or above the cut-off (i.e., the child is on track) [18].

#### 2.2.6. COVID-19 Questionnaire

The COVID-19 questionnaire consisted of 25 questions relating to the children’s physical activity, sedentary behavior, screen time, and sleep during the period of the COVID-19 restrictions in Sweden (i.e., May–June 2020). The questionnaire was divided into three sections: (i) background information on the child and participating parent (2 questions); (ii) physical activity, screen time, sedentary behavior, and sleep during the COVID-19 period (11 questions); and (iii) COVID-19 and its impact on your daily life (12 questions). The questions regarding time spent in various movement behaviors were identical to the previous questionnaire. Parents were sent a text message asking if they would be willing to participate and if they agreed, a telephone interview was booked so a member of the SUNRISE Sweden team who would call and conduct the questionnaire over the phone with them. This took on average 15 min.

### 2.3. Meeting the 24-Hour Movement Guidelines

To meet the WHO Global guidelines for preschool aged children (3–4 years) the following recommendations should be met: (i) preschoolers should be involved in at least 180 min of total physical activity (TPA) of which 60 min are spent in moderate to vigorous physical activity (MVPA) per day; (ii) screen time is no more than one hour per day; and (iii) sleep duration is between 10 to 13 h (including naps) [10].

### 2.4. Data Analysis

To evaluate the proportion of preschoolers meeting the WHO Global guidelines, descriptive statistics were calculated, where children were scored as either meeting or not meeting the guidelines separately and in combination. Differences between sexes were investigated using Wilcoxon’s rank-sum test and chi-squared test. All analyses were conducted in SPSS version 25 (IBM, Armonk, NY, USA) using a two-sided 5% level of significance.

## 3. Results

Table 1 shows the demographic characteristics and amount of time spent in the different movement behaviors. According to the WHO’s classification for BMI, 68 children were classified in the normal range and 32 children were classified as being at possible risk of overweight. In comparison to girls, boys participated in significantly more TPA (262.5 ± 60.3 vs. 220.4 ± 39.2 min, *p* = 0.002), MVPA (136.9 ± 43.8 vs. 107.8 ± 29.2 min, *p* = 0.004), and vigorous physical activity (39.0 ± 19.4 vs. 29.4 ± 15.7 min, *p* = 0.028). Girls spent significantly more time in sedentary behavior than boys (589.3 ± 47.3 vs. 544.7 ± 61.1 min, *p* = 0.002, respectively). No significant differences between sexes were found for the amount of time spent using screens or sleeping.

The proportions of children meeting the WHO Global guidelines are displayed in Table 2. Fourteen children (19.4%) met the components of the guidelines. When examining the guidelines separately, the screen time, physical activity, and sleep guidelines were met by 37.8% (*n* = 37), 90.3% (*n* = 65), and 62.5% (*n* = 45) of the children, respectively. No statistically significant differences between boys and girls were found for those meeting or not meeting the guidelines individually and in combination.

Table 3 displays the results of the executive function tasks. For the “Card Sort” and “Mr. Ant” tasks girls had higher scores than boys (6.39 ± 4.09 vs. 4.43 ± 4.13 points, respectively, *p* = 0.041 and (girls: 1.50 ± 0.95; boys: 1.13 ± 0.93, respectively). However, for the “Mr. Ant” task this result did not quite reach statistical significance (*p* = 0.055). No statistically significant difference between boys and girls was observed for the “Go/No Go” task.

The results of the gross and fine motor skills are presented in Table 4. For fine motor skills girls had higher scores than boys (39.76 ± 17.46 vs. 32.47 ± 17.64 points, respectively, *p* = 0.027). The proportion of all children who were developmentally on-track for the gross and fine motor skills were 52.0% and 33.0%, respectively. Table S1 shows the number and the proportion of children being able to perform each of the gross and fine motor skills individually.

During the COVID-19 pandemic the majority of Swedish parents reported that they were not concerned regarding their child’s physical activity (*n* = 77, 93.9%) and screen time (*n* = 73, 89.0%), as well as they felt they could support their child to have healthy movement behaviors (*n* = 80, 97.6%). Table 5 presents parental reported physical activity, time spent outside, and screen time pre-COVID-19 and during COVID-19. During the COVID-19 pandemic Swedish children’s physical activity, time spent outside on weekdays and weekend days, and screen time significantly increased (+53 min/day; +124 min/day; +68 min/day; +30 min/day, respectively; all *p*-values ≤ 0.001).

## 4. Discussion

Overall, a low proportion of Swedish preschool aged children met the WHO Global guidelines. The majority of the methods used in the SUNRISE study were found to be feasible in a Swedish context; however, low compliance for the ActiGraph protocol was found. Interestingly, some gender differences were found for some of the executive function tasks. Finally, the COVID-19 pandemic positively and negatively influenced Swedish preschool children’s physical activity and screen time, respectively.

### 4.1. Proportion of Children Meeting the WHO Global Guidelines

To the best of our knowledge, this is the first study in Sweden to investigate the number of preschool aged children meeting the WHO Global guidelines. A previous study by Berglind et al. [19] in Swedish preschool aged children has been conducted; however, they used the 24-Hour Movement Guidelines for children aged 5–17 years [20]. These guidelines differ significantly from those for the early years as these guidelines consist of: (i) ≥60 min of MVPA (180 min of TPA with at least 60 min spent in MVPA for the early years), (ii) ≤120 min of screen time (≤60 min for the early years), and (iii) 9–11 h of sleep per night (10–13 h for the early years) [10,20]. Thus, this makes the studies very difficult to compare.

The results obtained in the present study regarding the proportion of children meeting the WHO Global guidelines were slightly higher compared to previous studies from Canada [21], Australia [22], China [23], and Belgium [24] where 12.7%, 14.9%, 15.0%, and 5.6% met all three recommendations, respectively. However, in comparison to a study from Finland we found slightly lower proportions meeting all three guidelines (19.4% vs. 23.6%) [25]. Similar to our findings, Cliff et al. [22] and Leppänen et al. [25] found a high proportion of Australian and Finnish children meeting the physical activity recommendation (93.1% and 84.6%, respectively), whereas the other aforementioned studies had lower proportions ranging from 11.0% in Belgian preschoolers [24] to 65.4% in Chinese preschoolers [23]. It is important to highlight that that variation in time spent in various physical activity intensities could be partially attributed to methodological differences in the studies, as different monitors and cut-points were used. All the aforementioned studies used waist-worn ActiGraph accelerometers [22,23,24,25], except for the Canadian study [21], which used a waist-worn Actical monitor. However, for the studies using the ActiGraph accelerometer, including the present one, three different sets of cut-points were used. Thus, caution must be used when comparing results.

The low proportion of children meeting the screen time recommendation was also observed in Canada, Australia, Belgium, and Finland, where proportions ranged from 17.3% in Australia to 47.2% in Belgium [21,22,24,25]. With regards to sleep duration, we had a lower proportion of children meeting the sleep recommendation (62.5%) than Canada, Australia, Belgium, and Finland where 83.9%, 88.7%, 94.0%, and 75.7% met the recommendation, respectively [21,22,24,25]. However, we had a higher proportion than China where only 29.5% of children met the sleep recommendation [23]. Parental reported sleep collected via questionnaire was used in the Canadian [21], Australian [22], and Belgian [24] studies; whereas the Finnish [25] study used a 7-day sleep diary. In the present study we used objective measures of sleep using the ActiGraph accelerometer, which was also used in the Chinese study [23]. This could partially explain the lower proportion of children meeting the sleep recommendation, as parental reported sleep has been found to overestimate sleep duration in comparison to objective measures [26]. It is also important to note that the present study only reported nocturnal sleep; therefore, we could be slightly underestimating total sleep duration for some children.

### 4.2. Feasibility of the SUNRISE Protocol in a Swedish Context

The executive function tasks were well accepted by Swedish preschool children with only two children refusing to play at least one of the three executive function tasks. In comparison to preliminary norms in Australian children aged 4.0 to 4.4 years [17], Swedish children had a higher mean score for the Card Sort task (5.24 ± 4.20 vs. 3.87 ± 3.97), a similar mean score for the Go/No Go task (0.55 ± 0.25 vs. 0.54 ± 0.21), and a lower score for the Mr. Ant task (1.28 ± 0.95 vs. 1.57 ± 0.89). Interestingly, in the present study girls scored statistically significantly better than boys for the Card Sort task (*p* = 0.041) and the result was borderline statistically significant for the Mr. Ant task. To date, no study has investigated differences in the EYT toolbox tasks between genders. A recent study by Pan et al. [27] stated that gender has been reported on inconsistently in the literature with regards to executive function tasks, thus they aimed to explore this in their study. They found that females scored higher than males for cognitive flexibility, which is in line with the present study. However, it is important to note that their participants were older with a mean age of 11.12 years (range 6–16 years) compared to 4.0 years in the present study. Future studies with larger sample sizes are needed to investigate gender differences in executive function tasks starting in the early years.

The gross and fine motor skills tasks were feasible to perform within the Swedish preschool setting with only a small number of children refusing to preform specific tasks. In comparison to Norwegian, American, and Dutch children the Swedish children in the present study scored much lower for both gross and fine motor skills [28,29]. For example, Norwegian children’s average scores for the gross and fine motor skills were 54 ± 9 and 50 ± 13 points [28] compared to 41.7 ± 15.6 and 35.5 ± 17.9 points for Swedish children, respectively. The differences between the scores could be attributed to how the Ages and Stages Questionnaire was administered in all of the studies. The Ages and Stages questionnaire was developed as a screening tool during the developmental period, which was to be filled out by the parents. In the Norwegian, American, and Dutch studies [28,29], the questionnaires were filled out by the parents, whereas in the present study all tasks were performed by the children in front of trained data collectors. Thus, lower scores are to be expected in the current study as children were measured objectively compared to parental reported measures in the other studies. This is supported by a recent study by Zysset et al. [30] who found that parental reported motor skills in preschool children were only weakly associated with objectively measured motor skills. Therefore, future studies utilizing the Ages and Stages Questionnaire should consider having trained data collectors or health professionals administering the questionnaire in order to provide more reliable results.

We had low compliance with the ActiGraph protocol in the current study with 28 children not complying. Eight children refused to wear the ActiGraph at all, whereas the other 20 either refused to wear it at night or forgot to put it back on after taking it off. There have been many debates in the literature regarding wear compliance and the placement of accelerometers. Two studies in school aged children found greater wear compliance with wrist-worn monitors compared to waist-worn monitors [31,32]. Furthermore, the Mobile-based intervention intended to stop obesity in preschool-aged children (MINISTOP) randomized controlled trial found high wear compliance for the wrist-worn ActiGraph with 315 Swedish four year old children complying with the 24 h protocol at baseline and 288 of 300 children (i.e., 96.0%) adhering to the protocol at the 6-month follow-up [33]. Thus in order to increase compliance for the main SUNRISE study, considerations should be made to switch from a waist-worn monitor to a wrist-worn monitor.

### 4.3. COVID-19

According to Guan et al. [34], children usually accumulate their daily physical activity at school, including active transport to and from school, organized sports and activities, spending time at parks/playgrounds, and active play, whereas most sedentary behavior occurs in the home environment. In contrast to many countries during the COVID-19 pandemic, preschools, playgrounds, and parks in Sweden remained open and children’s organized sports and activities continued. The responsibility for social distancing was placed on Swedes themselves (or the parents of young children). Furthermore, preschools changed their routines to have children outside as much as possible. Thus, due to the aforementioned reasons, an increase in the children’s physical activity and time spent outside during the COVID-19 pandemic fits the Swedish environment at that time. Furthermore, a significant increase in Swedish preschoolers’ screen time was also observed during the COVID-19 pandemic (+30 min/day). This could be explained by the fact that children were kept home from preschool if they were symptomatic, thus providing them with an opportunity to be in front of screens more often. It is also important to note that children were one year older at the time of the COVID-19 questionnaire, so this could also have influenced the results.

### 4.4. Strengths, Limitations, and Implications

The SUNRISE Sweden pilot study has multiple strengths, which include the objective measures of physical activity, sleep, executive functions, as well as gross and fine motor skills. Furthermore, we find it unlikely that seasonal variations influenced the increase in physical activity and time spent outdoors as both assessment points were in the spring. As the COVID-19 study was undertaken soon after the start of the pandemic lockdown, it was not possible to validate any of the questions due to lack of time. However, it is important to note that the questions were agreed upon by the SUNRISE leadership group. Due to the fact that this was a pilot study, the ActiGraph protocol included only two 24 h periods (both weekdays), which may not provide an accurate overview of the children’s physical activity and sleep patterns. However, it is important to note that for the main SUNRISE study in Sweden the protocol will include seven measurement days. Another limitation of this study is that only nocturnal sleep from the ActiGraph accelerometer was taken into account, which could lead to an underestimation of total sleep in some children. However, due to the fact that parent-reported sleep is known to overestimate sleep duration [26], we found this to be a better measure. It is also relevant to note that the majority of Swedish children do not sleep during the daytime at this age. Finally, this study utilized convenience sampling from two regions in Sweden and coupled with the small sample size (*n* = 100), the generalizability of the results are limited. However, this was not the main aim of this study as this was a pilot study, with the primary aim to assess the feasibility of the measures for the main study, which will be conducted in a representative sample of Swedish preschool-aged children.

This study has several implications and suggestions for future research. First of all, a representative and dimensioned study regarding the proportion of Swedish children fulfilling the different components of the WHO Global guidelines is needed. Furthermore, our results indicate that a large proportion of Swedish children have excess screen time. Thus, a topic for future research is to investigate the underlying reasons and ways to reduce screen time in this age group. Our Swedish pilot study also provides valuable insights regarding the wear placement of the accelerometer for Sweden and other similar countries participating in SUNRISE as discussed above. Finally, this is one of the first studies to report on the changes in movement behaviors in a preschool population during the COVID-19 pandemic and provides important information for second or third waves of COVID-19 or future pandemics.

## 5. Conclusions

Just over 19% of Swedish preschool aged children met the WHO Global guidelines in this pilot study; however, larger studies with a representative sample size are needed to confirm or contrast these results. Children in this study had excessive screen time, thus future studies need to investigate the underlying reasons in order to create effective interventions to target this unhealthy behavior. Furthermore, we found that the executive function tasks as well as the gross and fine motor skill assessments were feasible in a Swedish context. Low compliance for the ActiGraph protocol was observed, with 20% not wearing the monitor overnight. Therefore, considerations should be made to switch to a wrist-worn monitor for the main SUNRISE study. Finally, the children’s physical activity and time spent outside actually increased during the pandemic, which is probably due to how the rules/restrictions were implemented in Sweden.

## Figures and Tables

**Table 1:** Descriptive characteristics and time spent in movement behaviors of the participating children.

	All (*n* = 100) ^1^	Boys (*n* = 58) ^2^	Girls (*n* = 42) ^3^	*p*-Value ^4^
	Mean ± SD	Mean ± SD	Mean ± SD	
Age	4.0 ± 0.5	4.0 ± 0.4	4.0 ± 0.5	0.986
Weight (kg)	17.4 ± 2.1	17.7 ± 2.2	16.9 ± 1.9	0.106
Height (cm)	103.8 ± 5.0	104.4 ± 5.5	103.1 ± 4.1	0.274
BMI (kg/m^2^)	16.1 ± 1.2	16.2 ± 1.1	15.9 ± 1.2	0.047
TPA (min/day)	243.8 ± 55.8	262.5 ± 60.3	220.4 ± 39.2	0.002
MVPA (min/day)	124.0 ± 40.5	136.9 ± 43.8	107.8 ± 29.2	0.004
VPA (min/day)	34.8 ± 18.4	39.0 ± 19.4	29.4 ± 15.7	0.028
Sedentary behavior (min/day)	564.6 ± 59.4	544.7 ± 61.1	589.3 ± 47.3	0.002
Screen time (min/day)	104.2 ± 68.2	106.2 ± 72.6	101.5 ± 62.4	0.735
Sleep (min/day)	611.7 ± 38.2	612.0 ± 43.0	611.2 ± 31.9	0.869

Abbreviations: *n*, Number of children; SD, Standard deviation; BMI, Body mass index; TPA, Total physical activity; MVPA, Moderate-to-vigorous physical activity; VPA, Vigorous physical activity; ^1^ For all physical activity intensities and sleep *n* = 72 and for screen time *n* = 98; ^2^ For all physical activity intensities and sleep *n* = 40 and for screen time *n* = 57; ^3^ For all physical activity intensities and sleep *n* = 32 and for screen time *n* = 41; ^4^ Differences between sex tested using Wilcoxon’s rank-sum test.

**Table 2:** Number and proportion of children meeting each of the 24-h movement guidelines stratified by sex ^1^.

	Total, *n* (%) ^1^	Boys, *n* (%) ^2^	Girls, *n* (%) ^3^	*p*-Value ^4^
≥60 min MVPA per day	71 (98.6%)	40 (100.0%)	31 (96.9%)	0.260
≥180 min TPA per day	65 (90.3%)	37 (92.5%)	28 (87.5%)	0.477
≥60 min MVPA and ≥180 min TPA per day	65 (90.3%)	37 (92.5%)	28 (87.5%)	0.477
≤60 min screen time per day	37 (37.8%)	22 (38.6%)	15 (36.6%)	0.839
10–13 h sleep per day	45 (62.5%)	24 (60.0%)	21 (65.6%)	0.624
Meeting all recommendations	14 (19.4%)	6 (15.0%)	8 (25.0%)	0.287

Abbreviations: *n*, Number of children; MVPA, Moderate-to-vigorous physical activity; TPA, Total physical activity; ^1^ All children: *n* = 72 for MVPA, TPA, sleep, and meeting all recommendations and *n* = 98 for screen time and sleep; ^2^ Boys: *n* = 40 for MVPA, TPA, sleep, and meeting all recommendations and *n* = 57 for screen time; ^3^ Girls: *n* = 32 for MVPA, TPA, sleep, and meeting all recommendations and *n* = 41 for screen time; ^4^ Differences between sexes tested using Pearson chi-square test.

**Table 3:** Scores on the executive function tasks (mean ± standard deviation).

	All (*n* = 99) ^1^	Boys (*n* =58)	Girls (*n* = 41)	*p*-Value ^3^
Card Sort ^1^	5.24 ± 4.20	4.43 ± 4.13	6.39 ± 4.09	0.041
Go/No Go ^1,2^	0.55 ± 0.25	0.53 ± 0.26	0.57 ± 0.24	0.435
Mr. Ant ^1^	1.28 ± 0.95	1.13 ± 0.93	1.50 ± 0.95	0.055

^1^ One child refused to play all three executive function tasks therefore *n* = 99 for all children and n = 41 for girls; ^2^ One child refused to play Go/No Go therefore *n* = 98 for all children and *n* = 57 for boys; ^3^ Differences between sex tested using Wilcoxon’s rank-sum test.

**Table 4:** Overall scores for the gross and fine motor skill (mean ± standard deviation).

	All (*n* = 100)	Boys (*n* =58)	Girls (*n* = 42)	*p*-Value ^1^
Gross motor skills	41.70 ± 15.59	40.66 ± 16.79	43.14 ± 13.85	0.607
Fine motor skills	35.53 ± 17.85	32.47 ± 17.64	39.76 ± 17.46	0.027

^1^ Differences between sex tested using independent *t*-test.

**Table 5:** Mean and standard deviation for parent reported physical activity, time spent outside, and screen time pre-COVID-19 and during COVID-19.

	Pre-Covid-19	During COVID-19	*p*-Value ^3^
Physical activity (min/day) ^1^	209 ± 119	262 ± 123	0.001
Time spent outside weekdays (min/day) ^2^	196 ± 85	320 ± 112	<0.001
Time spent outside weekend days (min/day) ^1^	191 ± 82	259 ± 96	<0.001
Screen time (min/day) ^1^	106 ± 71	136 ± 78	<0.001

^1^*n* = 81, one child was missing the movement behavior questions pre-COVID-19; ^2^ *n* = 78, three children missing pre-COVID-19 and one child missing during COVID-19; ^3^ Differences pre and during COVID-19 tested using Wilcoxon’s rank-sum test.

## Data Availability

The datasets supporting the conclusions of this article are available upon request to. Christine Delisle Nyström (christine.delisle.nystrom@ki.se).

## Supplementary Materials

The following are available online at https://www.mdpi.com/1660-4601/17/22/8491/s1, Table S1: Number and percentage of children being able to perform the gross and fine motor skills tasks.
