# Supplementary material for: International Study of Movement Behaviors in the Early Years (SUNRISE): Results from SUNRISE Sweden’s Pilot and COVID-19 Study
Source: Int J Environ Res Public Health. 2020 Nov 16;17(22):8491. doi: 10.3390/ijerph17228491 (PMC7698175; doi:10.3390/ijerph17228491)
Supplement: Supplementary file 1 [file ijerph-17-08491-s001.pdf]

Table S1. Number and percentage of children being able to perform the gross and fine motor skills tasks.

	All (n = 100)			Boys (n = 58)			Girls (n = 42)		
	**Yes**	**Sometimes**	**Not yet**	**Yes**	**Sometimes**	**Not yet**	**Yes**	**Sometimes**	**Not yet**
Gross									
Catching ball	34 (34.0%)	35 (35.0%)	31 (31.0%)	22 (37.9%)	14 (24.1%)	22 (37.9%)	12 (28.6%)	21 (50.0%)	9 (21.4%)
Throwing ball	82 (82.0%)	10 (10.0%)	8 (8.0%)	48 (82.8%)	6 (10.3%)	4 (6.9%)	34 (81.0%)	4 (9.5%)	4 (9.5%)
Hop on one leg	84 (84.0%)	1 (1.0%)	15 (15.0%)	46 (79.3%)	1 (1.7%)	11 (19.0%)	38 (90.5%)	0 (0%)	4 (9.5%)
Two-foot jump[1]	57 (57.6%)	10 (10.1%)	32 (32.3%)	32 (56.1%)	6 (10.5%)	19 (33.3%)	25 (59.5%)	4 (9.5%)	13 (31.0%)
Balance	56 (56.0%)	13 (13.0%)	31 (31.0%)	33 (56.9%)	4 (6.9%)	21 (36.2%)	23 (54.8%)	9 (21.4%)	10 (23.8%)
Fine									
Puzzle	52 (52.0%)	-	48 (48.0%)	30 (51.7%)	-	28 (48.3%)	22 (52.4%)	-	20 (47.6%)
Cutting[1]	61 (61.6%)	-	38 (38.4%)	30 (52.6%)	-	27 (47.4%)	31 (73.8%)	-	11 (26.2%)
Shapes[1]	78 (78.8%)	5 (5.1%)	16 (16.2%)	44 (77.2%)	2 (3.5%)	11 (19.3%)	34 (81.0%)	3 (7.1%)	5 (11.9%)
Unbutton	54 (54.0%)	-	46 (46.0%)	26 (44.8%)	-	32 (55.2%)	28 (66.7%)	-	14 (33.3%)
Draw[2]	72 (73.5%)	4 (4.1%)	22 (22.4%)	38 (67.9%)	3 (5.4%)	15 (26.8%)	34 (81.0%)	1 (2.4%)	7 (16.7%)
Colouring	52 (52.0%)	-	48 (48.0%)	27 (46.6%)	-	31 (53.4%)	25 (59.5%)	-	17 (40.5%)

[1] One child refused to perform the two-foot jump, cut, and copy the shapes therefore n = 99 for all children and n = 57 for boys

[2] Two children refused to draw the person therefore n = 98 for all children and n = 56 for boys
